# MHC Class II-Restricted Presentation of the Major House Dust Mite Allergen Der p 1 Is GILT-Dependent: Implications for Allergic Asthma

**DOI:** 10.1371/journal.pone.0051343

**Published:** 2013-01-11

**Authors:** Laura Ciaccia West, Jeff E. Grotzke, Peter Cresswell

**Affiliations:** Department of Immunobiology, Howard Hughes Medical Institute, Yale University School of Medicine, New Haven, Connecticut, United States of America; Ludwig-Maximilians-University Munich, Germany

## Abstract

Gamma-interferon-inducible lysosomal thiol reductase (GILT) is known to reduce disulfide bonds present in proteins internalized by antigen presenting cells, facilitating optimal processing and presentation of peptides on Major Histocompatibility Complex class II molecules, as well as the subsequent activation of CD4-positive T lymphocytes. Here, we show that GILT is required for class II-restricted processing and presentation of immunodominant epitopes from the major house dust mite allergen Der p 1. In the absence of GILT, CD4-positive T cell responses to Der p 1 are significantly reduced, resulting in mitigated allergic airway inflammation in response to Der p 1 and house dust mite extracts in a murine model of asthma.

## Introduction

Adaptive immune responses begin when antigen-presenting cells (APCs) become activated and internalize immunogenic proteins, which are then processed into peptides in endocytic compartments and presented on Major Histocompatibility Complex class II molecules (MHCII) to CD4-positive T lymphocytes. Optimal processing of an antigenic protein into presentable peptides requires acidification of the endocytic pathway [1], activity of endocytic proteases [2], and, if the original internalized protein contains disulfide bonds, reduction of these bonds by gamma-interferon-inducible lysosomal thiol reductase (GILT) [3]. In the absence of GILT, the only known thiol reductase in the endocytic compartment [4], epitopes that require disulfide bond reduction are lost, which results in a reduction in T cell stimulation, an effect demonstrated using model antigens, including hen egg lysozyme [5] as well as melanocyte differentiation antigens expressed by melanomas [6], [7], [8]. GILT also plays a role in Major Histocompatibility Complex class I (MHCI)-restricted cross-presentation of disulfide-containing antigens; for example, it is necessary for generation of the dominant H2-K^b^-restricted epitope derived from glycoprotein B expressed by herpes simplex virus 1 [9].

GILT is constitutively expressed in APCs and can be induced by IFN-γ in many cell types, a STAT1-dependent effect [10]. It is synthesized in the endoplasmic reticulum as a 35 kDa precursor and targeted to the endocytic pathway by the addition of mannose-6-phosphate (M6P), where it is cleaved at both the N- and C-termini by cathepsins to yield a 28 kDa mature form [11]. Both the precursor and mature forms of GILT have thiol reductase activity, which is mediated by a thioredoxin-like CXXC motif in the active site; this activity is optimal at the acidic pH of the lysosome but maintained at neutral pH [4].

Because endocytic GILT is required for robust CD4^+^ T cell responses, we reasoned that it might also be important in the generation of allergen-specific CD4^+^ T cells during allergic asthma responses. Many common allergens contain multiple disulfide bonds (Table 1) and thus likely require GILT for optimal endocytic processing and presentation on MHCII. Furthermore, because CD4^+^ T cells play a central role in asthma pathogenesis [12], we hypothesized that Gilt^−/−^ animals would display lessened asthmatic symptoms following intranasal challenge with a GILT-dependent allergen.

**Table 1:** Other asthma-related allergens contain disulfide bonds and thus may require GILT for optimal MHC class II-presentation.

NAME	DESCRIPTION	DISULFIDE BONDS
Bla g 2	Cockroach allergen (aspartic protease) [28]	5*
Bla g 4	Cockroach allergen (lipocalin) [29]	2
Der f 1	HDM allergen (*D. farinae*) [14]	3
Der p 1	HDM allergen (*D. pteronyssinus*) [14]	3
Fel d 1	Housecat allergen [30]	3

* Crystal structures and structural analysis of listed allergens were obtained from corresponding references.

Here, we demonstrate a role for GILT in antigen processing of and T cell responses to Der p 1, a major house dust mite (HDM) allergen that contains multiple disulfide bonds [13], [14] and is the most common asthma-associated allergen worldwide [15]. In the absence of GILT, we observed decreased Der p 1-specific CD4^+^ T cell responses due to deficient antigen processing of Der p 1 by GILT^−/−^ dendritic cells. Notably, this defect correlates with a mitigated allergic response in a mouse model of Der p 1-induced allergic asthma, suggesting that GILT can directly influence the allergic response by contributing to the endocytic processing of an allergen.

## Materials and Methods

### Animals

C57BL/6 (wild type (WT)) were purchased from The Jackson Laboratory (Bar Harbor, ME). GILT-deficient mice were generated as previously described [5]. Six- to 10-week old sex-matched mice were used in airway and recall proliferation experiments. Twelve-week old sex-matched mice were used to generate bone marrow-derived dendritic cells (DCs). This study was carried out in strict accordance with the recommendations in the Guide for the Care and Use of Laboratory Animals in the National Institutes of Health. The protocol was approved by the Institutional Animal Care and Use Committee at Yale University (protocol number: 2010-07721). All efforts were made to minimize suffering.

### Recall proliferation assays

Mice were injected subcutaneously at the base of tail with 100 µg of purified Der p 1 (Indoor Biotechnologies) or papain (Invitrogen), or 50 µg purified Bla g 2 or Der f 1 (Indoor Biotechnologies), in complete Freund's adjuvant (CFA). After ten days, the draining lymph nodes were removed and harvested. Cells were incubated with a range of Der p 1, Der p 1 peptide, papain, Bla g 2, or Der f 1 concentrations for 72 hours prior to addition of [^3^H]-thymidine. After an additional 24 hours, cells were harvested and [^3^H]-thymidine incorporation was assessed. For some experiments, lymph node cells were depleted of CD4^+^ or CD8^+^ T cells by negative selection using biotinylated antibodies to CD11b, CD11c, CD19, CD45R, CD49b, CD105, MHC class II, Ter-119 and CD8a or CD4, followed by magnetic, anti-Biotin microbeads (Miltenyi Biotec).

### Generation of DCs and co-culture with CD4^+^ T cells

Bone marrow-derived DCs were prepared from wild-type or Gilt^−/−^ mice. Bone marrow cells harvested from the femurs and tibiae of 8–10 week old mice were cultured for 5–7 days in RPMI 1640 supplemented with 10% fetal calf serum (Hyclone), 100 units/mL penicillin and 100 µg/mL streptomycin (GIBCO), 10 mM HEPES (GIBCO), 1% non-essential amino acids (GIBCO), 2 mM L-Glutamine (GIBCO), 1 mM sodium pyruvate (GIBCO), 0.025% β-mercaptoethanol (Sigma), and 20 ng/mL GM-CSF (R&D Systems). Resulting DCs were pulsed for 8 hours with various concentrations of Der p 1 or papain, and co-incubated with CD4^+^ T cells. To generate antigen-specific CD4^+^ T cells, wild-type C57BL/6 mice were injected subcutaneously at the base of the tail with 100 µg Der p 1 or papain emulsified in CFA. Draining lymph nodes were isolated ten days later, and CD4^+^ T cells were negatively selected using biotinylated antibodies to CD11b, CD11c, CD19, CD45R, CD49b, CD105, MHC class II, Ter-119 and CD8a, followed by magnetic, anti-Biotin microbeads (Miltenyi Biotec). After 72 hours incubation with DCs T cell proliferation was assessed using [^3^H]-thymidine as above.

### Airway challenge and BAL analysis

In some experiments mice (WT C57BL/6 and Gilt^−/−^ C57BL/6) were sensitized intranasally with 2 µg Der p 1 or papain and 0.1 µg lipopolysaccharide (LPS) in 20 µL phosphate-buffered saline (PBS) or 0.1 µg LPS in 20 µL PBS (negative control) on days 0 and 1. Mice were then challenged intranasally with identical doses on days 12 and 13, and sacrificed on day 14 for BAL analysis. BAL inflammatory cells were collected by cannulation of the trachea and lavage of the airway lumen with 1.2 mL of 5% fetal bovine serum in PBS. Live cells were counted based on Trypan-blue exclusion. In other experiments experimental animals were intranasally challenged with 100 µg HDM extract (Greer) dissolved in PBS while control animals were intranasally challenged with PBS alone. To evaluate eosinophilia, cytospin preparations were stained with Kwik-Diff (Thermo Scientific) and eosinophils were identified based on morphology and staining characteristics.

### qRT-PCR analysis

Following collection of BAL, the left lobe of the lung was removed, snap frozen in liquid nitrogen, and stored at −80°C. RNA was obtained from lung samples using the RNeasy Plus Mini Kit (Qiagen) and converted to cDNA with the High Capacity cDNA Reverse Transcription Kit (Applied Biosystems). To quantify expression of the cytokines IL-5, IL-13, and IFN-γ, as well as mucin-5AC, qRT-PCR was performed on a Stratagene Mx3000P using SYBR Green (Applied Biosystems) for product quantification. Signals were normalized to tubulin expression followed by normalization to PBS-treated controls using the comparative Ct method. Primer sequences were as follows (listed 5′ → 3′): IL-5 F-AGGCTTCCTGTCCCTACTCA, R-CTCCTCGCCACACTTCTCTT; IL-13 F-CCAATTGCAATGCCATCTAC, R-GCGAAACAGTTGCTTTGTGT; IFN-γ F-AGCTCTTCCTCATGGCTGTT, R-ATCTGGCTCTGCAGGATTTT; Muc5ac F-GACCAGCCATAGGGTTCACT, R-AACCCTCTTGACCACCTGAC; tubulin F-CAGGACGGCATCCACTAACT, R- GATCTTTCGGCCAGACAACT

## Results

### The Der p 1-specific CD4^+^ T cell response is reduced in Gilt^−/−^ mice

**Figure 1:**
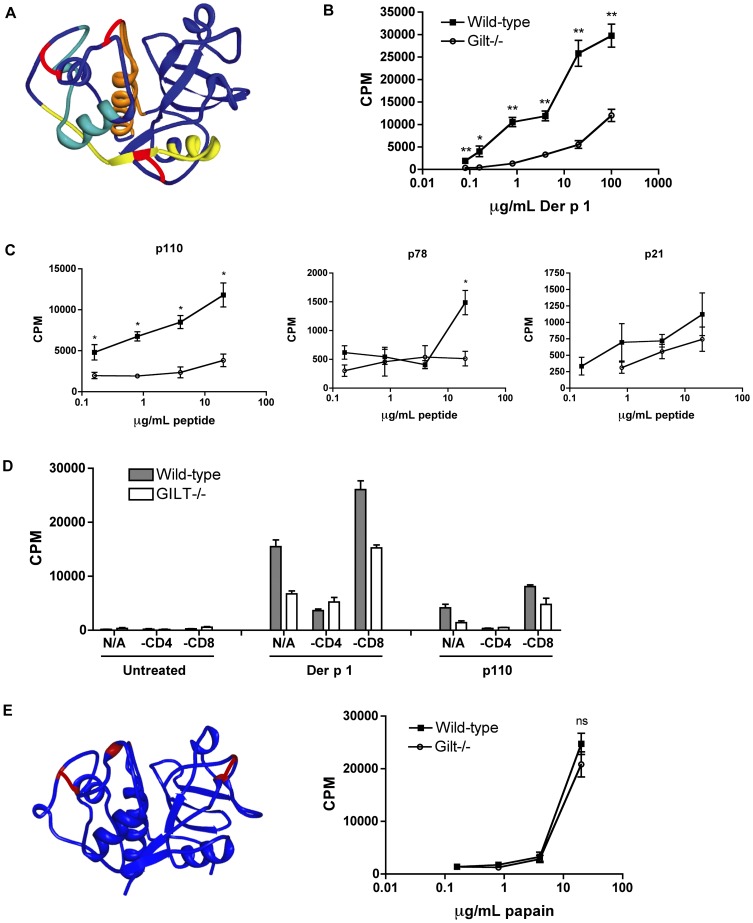
GILT^−/−^ lymphocytes exhibit deficient recall proliferation to Der p 1. *A*, Crystal structure of Der p 1 adapted from Chruszcz *et al* [14]. Disulfide bonds are shown in red; immunodominant epitopes are shown in yellow (p110), cyan (p78), and orange (p21). *B* and *C*, Recall proliferation analysis of lymphocytes isolated from Der p 1-challenged mice and incubated with increasing concentrations of purified Der p 1 (*B*) or immunodominant peptides (*C*). Data shown as mean ± SEM of triplicate wells and are representative of three independent experiments; * p<0.05, ** p<0.001. *D*, Recall proliferation analysis of lymphocytes from Der p 1-challenged mice that were depleted of CD8^+^ or CD4^+^ T cells prior to incubation with increasing concentrations of purified Der p 1 or p110 peptide. Data shown as mean ± SEM of triplicate wells and are representative of two independent experiments. *E*, Crystal structure of papain adapted from Kamphuls *et al* [19] and recall proliferation analysis of lymphocytes isolated from papain-challenged mice and incubated with increasing concentrations of papain. Data shown as mean ± SEM of triplicate wells and are representative of three independent experiments; p>0.05 for all points.

Previous work has characterized both the structure of Der p 1 [13], [14] and its immunodominant CD4^+^ epitopes [7]. A cysteine protease allergen produced by the house dust mite species *Dermatophagoides pteronyssinus*, it contains three disulfide bonds, two of which are proximal to or contained within the identified immunodominant I-A^b^-restricted epitopes p110 and p21 (Fig. 1*A*). This makes it a likely candidate for GILT-dependence. We therefore carried out a series of recall proliferation assays: first, wild-type and Gilt^−/−^ mice were injected subcutaneously at the base of the tail with 100 µg purified Der p 1 emulsified in CFA and allowed to rest for ten days. The draining lymph nodes (inguinal and sacral) were then isolated, and resultant lymphocytes were incubated with increasing amounts of purified Der p 1 (Fig. 1*B*) or synthetic peptides corresponding to the identified immunodominant Der p 1 I-A^b^ epitopes (Fig. 1*C*). After 72 hours, [^3^H]-thymidine was added, and cells were harvested after an additional 24 hour incubation. The recall response of the lymphocytes from the Gilt^−/−^ mice to Der p 1 was reduced by at least an order of magnitude compared to that of the wild-type lymphocytes (Fig. 1*B*). This effect was specific to Der p 1, as the recall response to the structurally related cysteine protease papain was identical for wild-type and Gilt^−/−^ lymphocytes (Fig. 1*E*). Furthermore, the Gilt^−/−^ lymphocytes demonstrated a dramatically reduced response to the immunodominant p110 peptide, which spans a disulfide bond, but a minimal difference in response to the p21 peptide, which does not (Fig. 1*C*). The response to the p78 peptide, which also contains a disulfide bond, was low in both cases, but again was reduced in the case of the Gilt^−/−^ mice.

We next sought to determine whether the observed effect was entirely due to reduced proliferation of CD4^+^ T cells, which would be consistent with a defect in MHCII processing. We repeated the Der p 1 recall proliferation assays after removing CD4^+^ T cells or CD8^+^ T cells from the lymphocyte populations prior to incubation with Der p 1 or p110. When CD4^+^ T cells were removed, the [^3^H]-thymidine incorporation was dramatically reduced in response to both Der p 1 and p110 (Fig. 1*D*). The removal of CD8^+^ T cells resulted in a comparatively enhanced proliferative response, likely due to an increase in the percentage of CD4^+^ T cells.

Overall, the data suggest that GILT contributes significantly to the endocytic processing of Der p 1 and its MHCII-restricted presentation to CD4^+^ T cells. In particular, GILT is required for a strong response to the p110 immunodominant epitope.

### The reduced recall response in Gilt^−/−^ mice results from a deficiency in Der p 1 antigen processing

**Figure 2:**
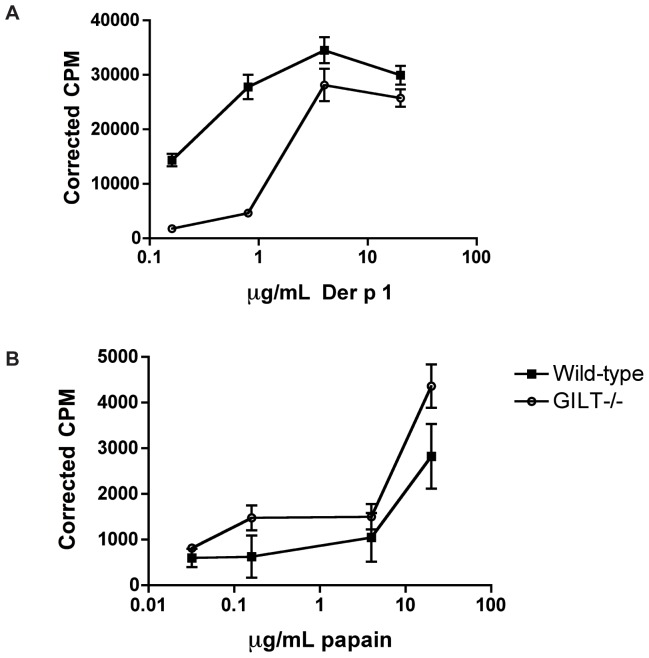
Wild-type CD4^+^ T cells exhibit deficient proliferation following co-incubation with Der p 1- but not papain-pulsed Gilt^−/−^ dendritic cells. *A*, Proliferation of CD4^+^ T cells isolated from Der p 1-challenged wild-type mice following a three day co-incubation with wild-type or Gilt^−/−^ bone marrow-derived dendritic cells that had been pulsed with increasing concentrations of purified Der p 1. *B*, Proliferation of CD4^+^ T cells isolated from papain-challenged wild-type mice following a three day co-incubation with wild-type or Gilt^−/−^ bone marrow-derived dendritic cells that had been pulsed with increasing concentrations of papain. Data shown as mean ± SEM of triplicate wells and are representative of at least two independent experiments.

Although the recall proliferation data (Fig. 1) suggest that GILT is involved in endocytic processing of Der p 1, we wanted to confirm that the difference in recall proliferation in the absence of GILT was due to defective antigen presentation rather than an inherent defect in the proliferating T cells. To address this question, we generated Der p 1-specific T cells by injecting wild-type mice subcutaneously with Der p 1 emulsified in CFA, isolating draining lymph nodes, and purifying CD4^+^ T cells from the resulting lymphocytes by negative selection. These T cells were then incubated with either wild-type or Gilt^−/−^ bone marrow-derived dendritic cells (DCs) that had been pulsed for 8 hours with purified Der p 1. After three days [^3^H]-thymidine was added to the cells, which were then harvested and ^3^H uptake assessed after an additional 24 hours. Thymidine incorporation was then normalized to that of CD4^+^ T cells that were incubated with Der p 1 alone. The sensitized wild-type CD4^+^ T cells proliferated significantly less well when stimulated by Gilt^−/−^ DCs than when co-incubated with wild-type DCs (Fig. 2*A*), which both supports the recall proliferation data and demonstrates that the observed deficiency in proliferation is due to defective antigen processing and presentation of Der p 1 in the absence of GILT. That purified CD4^+^ T cells from papain-challenged wild-type proliferate similarly when incubated with papain-pulsed wild-type or Gilt^−/−^ dendritic cells further supports this argument (Fig. 2*B*).

### Gilt^−/−^ mice develop less bronchoalveolar inflammation following intranasal Der p 1 challenge

**Figure 3:**
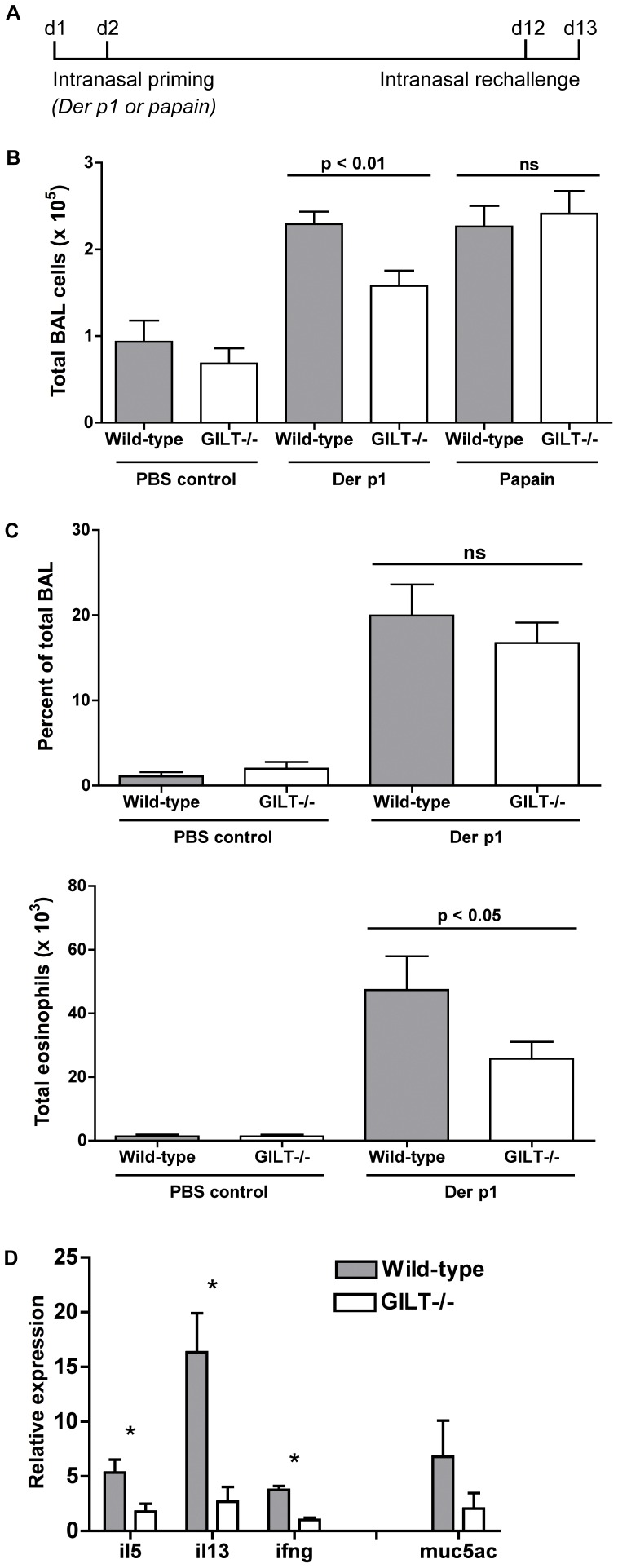
Gilt*^-/-^* mice develop reduced bronchoalveolar lavage cellularity following intranasal Der p 1 challenge. *A*, Schematic of the Der p 1/papain allergic asthma model. Mice were intranasally primed on day 0 and 1 with 0.1 µg LPS as Th2 adjuvant. Mice were rechallenged on day 12 and 13, then sacrificed 24 hours later for BAL analysis. *B*, BAL cellularity from age- and sex-matched wild-type and Gilt^−/−^ mice challenged with PBS, Der p 1, or papain. Pooled data from three to six mice from three (papain) or four (Der p 1) experiments are shown as mean ± SEM. *C*. Percentage of total BAL cellularity represented by eosinophils and total eosinophilia in Der p 1-challenged or control wild-type and Gilt^−/−^ mice. *D*. Relative expression of cytokines (IL-5, IL-13, IFN-γ) or MUC5AC in wild-type or Gilt^−/−^ lung tissue following intranasal Der p 1 challenge. Expression is normalized to β-tubulin expression and control samples. Data shown as mean ± SEM of samples from two to four mice and are representative of two experiments. Asterisks denotes p value less than 0.05.

Having established that GILT is involved in Der p 1 antigen processing and presentation, we wanted to determine whether it could affect the initiation and propagation of asthma symptoms. To address this question, we used an experimental model of allergic asthma: age- and sex-matched mice were challenged intranasally with purified Der p 1, following the scheme depicted in Fig. 3*A*. The mice were primed on days 1 and 2, rested, re-challenged on days 12 and 13, then sacrificed on day 14, when we measured cell accumulation in the bronchoalveolar lavage fluid (BAL). Gilt^−/−^ mice challenged with Der p 1 developed significantly (p<0.01) less total BAL cellularity (Fig. 3*B*) and less BAL eosinophilia (Fig. 3*C*) than wild-type mice. Notably, the percentages of total BAL cellularity made up by eosinophils (Fig. 3*C*), macrophages, neutrophils, and lymphocytes (data not shown) were comparable between both Gilt^−/−^ and wild-type animals, indicating that while the magnitude of the allergic airway response was diminished in the absence of GILT, the Th2 nature of the response was unaltered. Also unchanged was dendritic cell migration, which was measured by determining the average number CD163^+^ extravasating cells per blood vessel (data not shown).

We also performed qRT-PCR analysis of the lung tissue to evaluate the expression of pro-asthmatic factors, such as the Th2 cytokines IL-5 and IL-13. IL-5 is a key factor for eosinophil survival and IL-13 contributes to airway hyper-responsiveness and tissue remodeling in the lung [16]. We found significantly higher expression of both IL-5 and IL-13 in the lungs of wild-type mice (Fig. 3*C*), supporting the observation that the allergic airway response is curbed in Gilt^−/−^ mice. The characteristic Th1 cytokine IFN-γ was also expressed at lower levels in the lungs of Gilt^−/−^ mice (Fig. 3*C*), supporting the suggestion made above that the absence of GILT does not affect the characteristic Th2 nature of the allergic airway response. If it did, one might have expected higher expression of IFN-γ in the Gilt^−/−^ than in wild-type mice. We also measured the expression of mucin-5AC, a gel-producing protein that is upregulated during airway inflammation [17], and found that its levels were noticeably reduced in the absence of GILT.

These results suggest that GILT plays a role in the allergic airway response by reducing Der p 1 and facilitating its endocytic processing. We discovered that papain, a cysteine protease allergen [18] that is structurally related to Der p 1 [19], does not require GILT for optimal presentation, as measured by standard recall proliferation (Fig. 1*E*) and by DC-T cell co-culture (Fig. 2*B*), likely because the immunodominant epitopes can be generated independently of the oxidation state of the papain disulfide bonds. If the lung inflammation difference we observed for Der p 1 were due to GILT's role in endocytic processing, we would not expect to see such a difference if we substituted papain for Der p 1 as an inhaled allergen. Indeed, when we performed such an experiment, wild-type and Gilt^−/−^ mice exhibited similar cellular accumulation in the BAL after sensitization and inhalation of papain (Fig. 3*B*). This supports the hypothesis that GILT contributes to the development of HDM-induced asthma by facilitating presentation of Der p 1-derived antigens.

### Gilt^−/−^ mice develop reduced bronchoalveolar lavage cellularity and histological symptoms following intranasal house dust mite challenge

**Figure 4:**
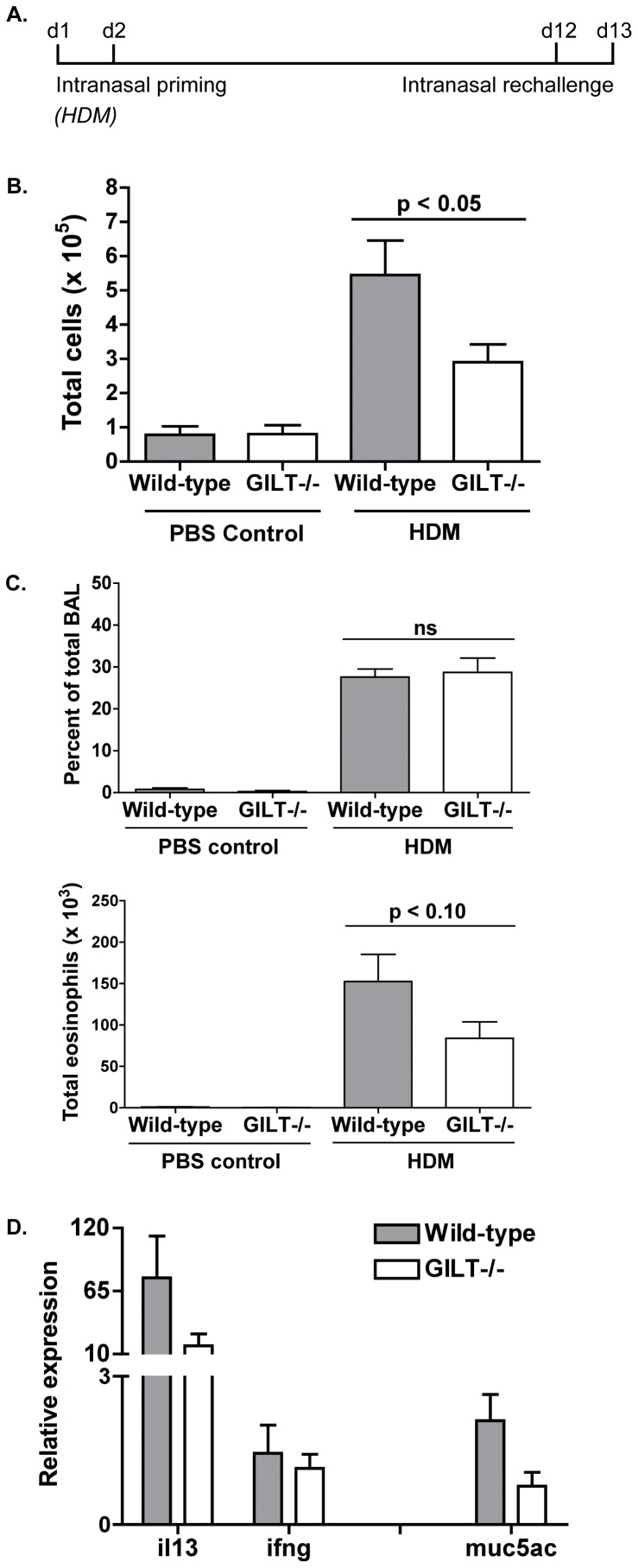
Gilt^−/−^ mice develop less bronchoalveolar lavage cellularity and histological symptoms following intranasal house dust mite (HDM) challenge. *A*, Schematic of the HDM asthma model used for BAL and histological analysis. Mice were intranasally primed on day 0 and 1 with 100–300 µg HDM extract and rechallenged on day 12 and 13 (BAL), then sacrificed 24 hours later for analysis. *B*, BAL cellularity from age- and sex-matched wild-type and Gilt^−/−^ mice. Pooled data from three to nine mice from two experiments are shown as mean ± SEM. *C*. Percentage of total BAL cellularity represented by eosinophils and total eosinophilia in HDM-challenged or control wild-type and Gilt^−/−^ mice. *D*. Relative cytokine expression (IL-13, IFN-γ) or MUC5AC in wild-type or Gilt^−/−^ lung tissue following intranasal HDM challenge. Expression is normalized to β-tubulin expression and control samples. Data shown as mean ± SEM of samples from two to nine mice and are averaged from two experiments.

Allergic asthma experiments using Der p 1 as the inhaled allergen demonstrated that Gilt^−/−^ mice develop a diminished response to Der p 1, as measured by BAL cellularity, eosinophilia, and expression of Th2 markers in the lungs. As inhalation of a purified allergen does not typically occur in physiological circumstances, we wanted to determine whether GILT also contributes to development of an allergic response to crude HDM extract. Age- and sex-matched mice were challenged intranasally with HDM extract, following the scheme depicted (Fig. 4*A*): the mice were primed on days 1 and 2, rested, re-challenged on days 12 and 13, then sacrificed on day 14 for analysis. As in the experiments using Der p 1, we measured total BAL cellularity, eosinophilia, and Th2 cytokine and mucin-5AC expression in the lungs to phenotypically evaluate HDM-induced allergic airway inflammation.

As observed with purified Der p 1, Gilt^−/−^ mice develop significantly less (p<0.05) BAL cellularity following intranasal challenge with HDM (Fig. 4*B*). Likewise, while the percentage of total BAL cellularity made up of eosinophils is similar between wild-type and Gilt^−/−^ animals, total eosinophilia is reduced in the absence of GILT (Fig. 4*C*), suggesting that the magnitude but not the Th2 character of response is reduced. This is supported by qRT-PCR analysis of HDM-challenged lung tissue, which showed decreased relative expression of the Th2 cytokine IL-13 and mucin-5AC in Gilt^−/−^ mice (Fig. 4*D*) but no difference in expression of the Th1 cytokine IFN-γ. Expression of IL-5 was not detectable in any samples from HDM-challenged mice, and was therefore not compared. Overall, while results are more variable due to the heterogeneity of HDM samples as compared to purified Der p 1, analysis of HDM-challenged Gilt^−/−^ mice suggests that the requirement of GILT for antigen processing of Der p 1 results in reduced lung inflammation in response to HDM inhalation.

### The CD4^+^ T cell response to the cockroach allergen Bla g 2 is also reduced in Gilt^−/−^ mice

To determine whether GILT might influence the MHCII-restricted response to additional asthma-linked allergens, indicating a potentially more general role for GILT in asthma, we performed recall proliferation assays using the cockroach allergen Bla g 2 [20] and the house dust mite allergen Der f 1, which both contain disulfide bonds (Table 1).

**Figure 5:**
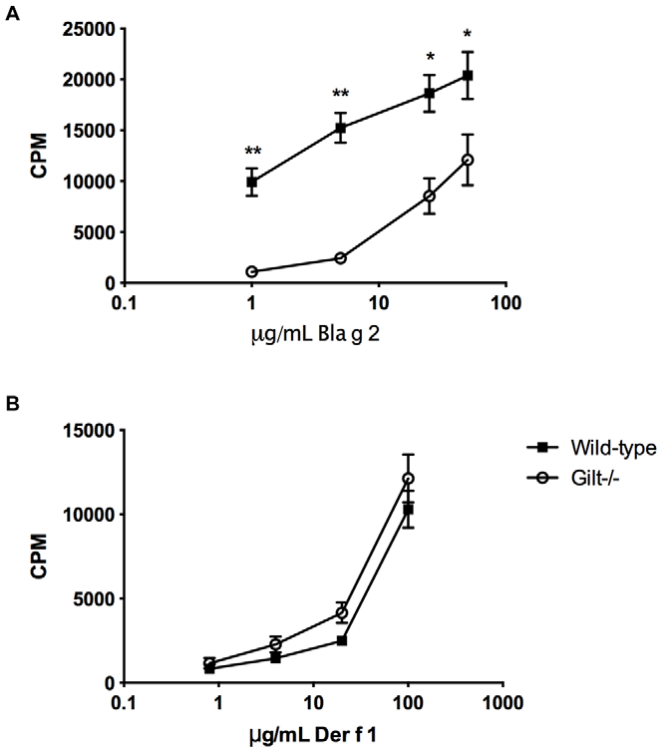
Gilt^−/−^ lymphocytes exhibit deficient recall proliferation to the cockroach allergen Bla g 2 but not to the house dust mite allergen Der f 1. Recall proliferation analysis of lymphocytes isolated from (*A*) Bla g 2- or (*B*) Der f 1-challenged mice and incubated with increasing concentrations of purified (*A*) Bla g 2 or (*B*) Der f 1. Data shown as mean ± SEM of triplicate wells and are representative of two independent experiments; * p<0.05, ** p<0.001.

Bla g 2, an aspartic proteinase that has five intrachain disulfide bonds [30], required GILT for optimal recall proliferation: Gilt^−/−^ lymphocytes isolated from mice injected subcutaneously with purified Bla g 2 exhibited a deficient recall response (Fig. 5*B*). This suggests that GILT-mediated endocytic reduction of Bla g 2 facilitates endosomal degradation and leads to the optimal presentation of its immunodominant CD4^+^ epitopes. Because exposure to Bla g 2 is linked to allergic asthma, GILT may contribute to cockroach-induced lung inflammation, as it does for HDM-induced lung inflammation.

While Der p 1 and Bla g 2 required GILT activity for optimal MHC class II-restricted responses, Der f 1, which is structurally related to Der p 1, did not (Fig. 5*B*). As noted above, the recall response to papain, which is also structurally related, was also GILT-independent (Figs. 1*E*, 2*B*). The differences in GILT-dependence are likely determined by the individual CD4+ T cell epitopes selected during the *in vivo* response. Sequence comparison of Der f 1 and Der p 1, which are structurally identical and share greater than 80% sequence homology [14], reveals that the region encompassing the immunodominant GILT-dependent Der p 1 epitope p110 differs by six amino acids in Der f 1, while the GILT-dependent minor epitope p78 differs by five. Interestingly, the p21 epitope, which is essentially GILT-independent, is identical in sequence in Der f 1 and Der p 1. Differences in the immunodominant epitopes and their dependence on GILT likely explain why GILT is needed for MHC class II-restricted presentation of some, but not all, allergens. A corollary of this finding is that the GILT-dependence of the response to individual allergens is likely to depend on the MHC class II alleles expressed. The studies reported here are restricted to H2^b^ mice, which express only a single MHC class II allele, I-A^b^.

## Discussion

Allergic asthma is initiated when allergens such as Der p 1 are inhaled into the airways and internalized by lung-resident APCs, which then process and present these allergens by MHCII to CD4-positive T cells. Because they orchestrate the Th2 response that characterizes asthma, including the production of type 2 cytokines, mucus hypersecretion, and IgE over-production, activation of CD4^+^ T cells by APCs is a key step in the progression of asthma symptoms [12].

Here, we show that GILT is an important factor for optimal MHCII presentation of the allergen Der p 1. In the absence of GILT, we observed decreased recall proliferation of lymphocytes in response to Der p 1, a deficiency shown to be due to impaired presentation of Der p 1-derived immunodominant epitopes by Gilt^−/−^ APCs. GILT likely contributes to the endocytic processing of Der p 1 by reducing one or more of its three disulfide bonds, a structural feature Der p 1 shares with several other asthma-linked allergens (Table 1). For this reason, GILT may be important for the presentation other disulfide-containing, asthma-linked allergens, as we have shown for the asthma-linked cockroach allergen Bla g 2. Whether GILT is required for optimal MHC class II-restricted presentation depends on the location of immunodominant epitopes in relation to the protein's disulfide bonds, probably explaining the GILT-independence of the overall CD4^+^ T cell responses to papain and Der f 1.

Due to its contribution to allergen processing and presentation on MHCII, GILT contributes to the initiation and progression of asthma in a mouse model of allergic lung inflammation. Following intranasal challenge with purified Der p 1, Gilt^−/−^ animals accumulate on average 30% less total cellularity and eosinophilia in the BAL and exhibit decreased expression of mucin-5AC and the Th2 cytokines IL-5 and IL-13 in the lung, suggesting a reduced allergic airway response to Der p 1. Furthermore, intranasal challenge with the more physiologically relevant crude HDM extract results in significantly less total cellularity in the BAL of Gilt^−/−^ mice, supporting the hypothesis that GILT plays a role in the allergic response by facilitating endocytic processing and MHCII presentation of allergen-derived antigens. Although differences in eosinophilia and IL-13 and mucin-5AC expression using this model failed to reach statistical significance, we believe this is likely due to the inherent heterogeneity of house dust mite extracts, which contain variable levels of LPS, chitins, and other allergens and can therefore elicit more complex immune responses than those to Der p 1 alone [21]. Overall, our data support the hypothesis that GILT contributes to HDM-induced lung inflammation by facilitating the antigen presentation of the allergen Der p 1.

It is possible that GILT may play an additional role in initiating and propagating the allergic response, as GILT has other known functions apart from its involvement in antigen processing. For example, intracellular GILT acts as a critical host factor for *Listeria monocytogenes* by enzymatically reducing and activating listeriolysin O [22], and it also contributes to cellular redox state by promoting the stability of superoxide dismutase 2 [23]. Interestingly, enzymatically active GILT is secreted by inflammatory macrophages [24], [25]. GILT levels in the BAL doubled following intranasal challenge with HDM (data not shown), suggesting that GILT might have extracellular effects during asthma as well. It is possible that GILT could affect the allergic response by altering the oxidative state of an important factor or by indirectly influencing the oxidative state of the lung [26]: for example, decreases in the level of reduced thiols in the lungs have been linked with disease severity in asthmatic children [27]. If either of these possibilities were true, this could also explain the large sample-to-sample variability in the allergic response to HDM observed in the experiments reported here: GILT could be affecting processes beyond antigen presentation of Der p 1.
